# External relationships as implementation determinants in community-engaged, equity-focused COVID-19 vaccination events

**DOI:** 10.3389/frhs.2024.1338622

**Published:** 2024-03-11

**Authors:** Ramey Moore, Jennifer Callaghan-Koru, Jennifer L. Vincenzo, Susan K. Patton, Marissa J. Spear, Sheldon Riklon, Eldon Alik, Alan Padilla Ramos, Stephanie Takamaru, Pearl A. McElfish, Geoffrey M. Curran

**Affiliations:** ^1^ Office of Community Health and Research, College of Medicine, University of Arkansas for Medical Sciences Northwest, Springdale, AR, United States; ^2^ Geriatrics, College of Health Professions, University of Arkansas for Medical Sciences Northwest, Fayetteville, AR, United States; ^3^ Nursing, College of Education and Health Professions, University of Arkansas, Fayetteville, AR, United States; ^4^ Office of Community Health and Research, University of Arkansas for Medical Sciences Northwest, Springdale, AR, United States; ^5^ Department of Family Medicine, Family Medicine Residency Training Program, College of Medicine, University of Arkansas for Medical Sciences Northwest, Fayetteville, AR, United States; ^6^ Consulate General of Arkansas, Republic of the Marshall Islands, Springdale, AR, United States; ^7^ Arkansas Coalition of Marshallese, Springdale, AR, United States; ^8^ Department of Pharmacy Practice, College of Pharmacy, University of Arkansas for Medical Sciences, Little Rock, AR, United States; ^9^ Center for Mental Healthcare and Outcomes Research, Central Arkansas Veterans Healthcare System, North Little Rock, AR, United States

**Keywords:** health equity, external networks, community engagement, community-based implementation, implementation science

## Abstract

**Background:**

While relationships and connectedness among organizations have been included in implementation theories, models, and frameworks, the increased attention to health equity in implementation science raises the urgency of understanding the role of relationships external to the implementing organization. This paper addresses this gap through an exploration of the role of external relationships in community-based, equity-focused interventions.

**Methods:**

This study focuses on an equity-focused, community-based COVID-19 vaccination intervention in Arkansas, drawing upon long-term community-engaged relationships among University of Arkansas for Medical Sciences and the Hispanic and Marshallese Islander communities. We used an exploratory qualitative descriptive design to examine barriers and facilitators to implementation of COVID-19 vaccination events analyzing in-depth qualitative interviews with implementation team members (*n* = 17).

**Results:**

All participants described pre-existing relationships among the implementing organization, partner organizations, and communities as a key implementation determinant for this equity-focused program. At the inter-organizational level, external relationships included formal connections and informal relationships among staff (e.g., communication channels from prior partnerships). At the individual level, strong external relationships with the community were facilitators leveraging long-term engagement, community familiarity, and staff from the communities of focus. Strong external relationships facilitated program reach in underserved communities through three mechanisms: (1) reduced time required to establish functional working relationships among partners; (2) accessibility and cultural congruence of health services; and (3) increased trust among community members. Barriers to implementation also existed in external relationships, but had less influence than facilitators.

**Conclusions:**

Achieving health equity in implementation science requires greater understanding of external relationships as implementation determinants. This exploratory study makes a significant contribution to the literature by describing the types of external relationships that facilitate equitable implementation and identifying the mechanisms through which they may work. We argue that approaches to community engagement drawn from community-engaged research approaches may be useful, as these processes require investment in building/maintaining formal and informal organizational and interpersonal relationships. Further research is needed to understand connections among external relationships and other implementation determinants.

## Introduction

Relationships and connectedness among organizations within and across the implementation environment has been included in implementation-related theories, models, and frameworks prior to the inception of implementation science (IS) as a field of study in the United States (US) at the turn of the 21st century (1). Communication channels and social systems are two of the four main elements in Roger's Diffusion of Innovation theory (2), a pillar upon which IS rests. Organizational relationships are also reflected in more recent implementation determinant frameworks (3) as “external networks” (4), “interconnections/linkages” (5), and “inter-organizational networks & relationships” (6). Despite the recognition of external relationships as a critical determinant of implementation (7–12), this construct has not been studied with the same depth as many other constructs within IS frameworks (13).

In the original version of the Consolidated Framework for Implementation Research (CFIR), one of the most widely-cited implementation determinants frameworks, “the degree to which an organization is networked with other external organizations” is identified as a critical implementation factor (14). In the revised CFIR “2.0” (4), the “partnerships and connections” construct broadly captures relationships with external organizations. This construct is situated within CFIR's *Outer Setting* domain, which captures “macro-level” implementation factors emanating from outside the *Inner Setting*, or the site where implementation is occurring. Perhaps as a result of the highly interventional nature of implementation research in healthcare, more attention has been paid to the *Inner Setting* and specifically to constructs and determinants within this category (e.g., available resources, infrastructure, incentive systems) which may be modifiable (or leveraged) within projects designed to improve implementation of a specific intervention or practice *within* specific healthcare organizations/locations (3). Other implementation frameworks have provided alternate constructs to focus on macro-level implementation factors, such as “inter-organizational networks” and “community-academic partnerships,” in the Exploration, Preparation, Implementation, Sustainment (EPIS) framework (15) and “inter-organizational networks & relationships” in the integrated-Promoting Action on Research Implementation in Health Services (iPARIHS) framework (6).

The limited attention on relationships among implementing organizations and external partners may be due to a perception that they are a more remote implementation determinant and that they are less amenable to rapid intervention. As such, relationships among the implementing organization and organizations and communities external to the implementing organization are a determinant that remains relatively under-conceptualized, and to-date few scholars have explored this construct in depth (16). Underdevelopment is a noted challenge for all outer setting constructs, which are “notoriously difficult to evaluate and influence” (13). Extant implementation research frameworks have also predominantly focused on relationships among similar or peer organizations (e.g., healthcare organizations, social services providers) (5, 14). Most published studies assessing external relationships primarily focus on links among peer organizations, such as formal implementation networks (17), quality improvement collaboratives (18), or organizations providing similar client services (19–21). The updated definition of the “partnerships and connections” construct in CFIR 2.0 helps expand the scope of external relationships to include collaboratives, professional societies, referral networks, community-academic partnerships, advocacy groups, and technical assistance organizations (4). While it has been noted that relationships with community organizations in different sectors (e.g., churches, non-profits) can benefit implementation (22, 23), and intersectoral relationships are a common approach in public health programs (24–26), their role is understudied in IS.

### Community engagement and equitable implementation

The increased attention to health equity in IS raises the urgency of understanding external relationships (organizational and/or among individuals) as determinants of implementation (27, 28). It is well-recognized that communities with the highest burden of health disparities are often unreached, or the last to be reached by evidence-based interventions (29–31). The determinants of healthcare organizations' ability to reach disproportionately-impacted communities is understudied in IS (32). A key recommendation for advancing health equity in IS is to engage equity partners in sectors outside of health systems [e.g., employers, housing, school, and faith-based organizations (FBO)] (27). Yet, little is currently understood about the extent to which healthcare organizations are able to engage external equity partners in the implementation of interventions, how best to engage partner organizations, and how these external relationships might improve equity of implementation and outcomes. Thus, when and how healthcare organizations engage underserved communities and the degree of the connectedness among these organizations and communities may emerge as a critical determinant of equitable outcomes.

Relationships between implementing organizations and community organizations are not an explicit component of new, equity-oriented implementation research frameworks (33, 34). While activities to engage communities can be considered as an equity-focused implementation strategy, the nature and strength of external relationships with community groups will likely determine the success of this strategy (35, 36), although recent work by Wallerstein and colleagues highlights that the science has lagged behind practice (37). IS research can draw upon the rich literature on Community-Engaged Research (CEnR) to facilitate our understanding of the role for relationships among community-based equity partners and implementing organizations (4–8).

Trust is another underdeveloped concept in the IS literature but is recognized as critical to building implementation partnerships and to recipients' participation (38). Conceptualizations of trust in CEnR have highlighted how relational dynamics contribute to trust-building (39), and establishing partnerships with underserved communities builds trust in healthcare organizations (40). This is also critical for equity in vaccine uptake, as community-engagement has frequently been identified as a critical factor in effectively promoting vaccine uptake and building trust in public health authorities and interventions, especially among marginalized and underserved communities (41–45). Therefore, it is necessary to understand whether, and how, external relationships improve the equity of implementation, relationship strength, and trust.

This paper addresses the gap in research on external relationships in implementation exploring the role of these relationships in implementing an equity-focused COVID-19 vaccination program. We utilized an exploratory qualitative descriptive study design to understand the barriers and facilitators to implementation of COVID-19 vaccination events within FBOs as a way to reach Hispanic and Marshallese Islander (hereafter Marshallese) community members.

## Methods

### Setting and intervention

The focus of this paper is the implementation of a community-based COVID-19 vaccination program in Arkansas. This program drew upon long-term community-engaged relationships between University of Arkansas for Medical Sciences (UAMS) and the Hispanic and Marshallese communities in the region (46, 47). These relationships originated in 2013 to address social determinants of health and associated chronic disease disparities among the Hispanic and Marshallese communities in Arkansas (41, 46). Since its inception, these relationships have utilized a community-engaged approach, which seeks to build trust among academic researchers, healthcare providers, and communities through direct engagement, honoring those communities' unique contributions at all stages of health interventions. Further details are published elsewhere (46, 48).

As an extension of these formal and informal pre-existing, community-engaged relationships, a COVID-19 response taskforce was developed and led by community-based organizations, and the taskforce met weekly between March 2020 (within one week of the first identified case of COVID-19 in Arkansas) and continued to meet through August of 2022, with daily communication among organizations to address the COVID-19 health disparities among the Hispanic and Marshallese populations in Northwest Arkansas (the details of which have been previously published) (41). The taskforce developed a comprehensive COVID-19 response involving education, outreach, testing, contact tracing, and support for quarantining (46). Vaccination outreach was included as COVID-19 vaccines became available in December 2020. Leveraging these relationships, academic researchers and healthcare organizations implemented COVID-19 vaccination events in community settings, primarily in partnership with FBOs, with a goal of improving reach, increasing attendee comfort, and providing native-language facilitation and education.

To support vaccination outreach programs, the academic medical center, UAMS, received funding from the National Institutes of Health-funded Community Engagement Alliance Against COVID-19 Disparities (CEAL); Racial and Ethnic Approaches to Community Health (REACH), administered by the Centers for Disease Control and Prevention; and the Health Resources and Services Administration (HRSA) of the United States Department of Health and Human Services. To maximize accessibility of the events, most were held at local FBOs with Hispanic and/or Marshallese congregations on days and times chosen to facilitate attendance and reduce barriers (described in previous publications) (49). Community health workers affiliated with FBOs and/or UAMS promoted attendance by scheduling appointments and providing resources such as transportation to attendees. Events were staffed by members of the implementing and partner organizations. The implementation team included healthcare providers, program staff [many of whom were community health workers (CHWs)], and staff of FBOs. All vaccines were administered by clinical staff, and all events included bilingual (English/Spanish or English/Marshallese) team members who provided medical translation.

### Data collection

Our exploratory qualitative descriptive study examined the barriers and facilitators to implementation of COVID-19 vaccination events within FBOs as a way to reach Hispanic and Marshallese community members. Data was collected at vaccination events held between July 2021 and September 2021. For transparency, our diverse research team and co-authors' self-identified positionalities include five men, six women, six identifying their race/ethnicity as non-Hispanic White, three identifying as Marshallese and Pacific Islanders, one identifying as Hispanic, and one identifying as mixed-race and ethnicity. Three qualitative researchers (GC, JV, and SP) conducted five observations of vaccination events held in Hispanic FBOs (*n* = 2), Marshallese FBOs (*n* = 2), and one (*n* = 1) church-affiliated community space. The three qualitative researchers also conducted informal interviews during events (*n* = 55) and invited team members at vaccination events to participate in a semi-structured interview at a later date. Informal interviews consisted of short, unstructured conversations with team members concerning their experiences with vaccination events.

Following a purposive sampling approach (50), the study team recruited 17 participants reflecting diverse roles in the implementation of vaccination events, which follows standard qualitative approaches to determining sample sizes based on the scope and nature of the study (51, 52). Inclusion criteria for participation consisted of adults (≥18 years of age) who were members of the implementation team. Formal, semi-structured qualitative interviews were conducted with participants via secure video conferencing in the fall of 2021. All interviews were conducted in English, transcribed verbatim, and de-identified before analysis. Verbal consent was obtained prior to interviewing and recorded in REDCap, along with demographic information (53, 54). Most interviews lasted between 30 and 60 min, and participants were provided a $50 incentive.

We used a semi-structured interview guide combining grand tour, open-ended questions, probe questions based on *a priori* CFIR categories, and topics emerging from informal interviews and observations at vaccination events to maintain consistency across formal interviews. The CFIR framework was chosen due to its comprehensive focus on implementation determinants and its frequent utilization within the IS literature. Examples of grand tour questions include, “What do you think worked well at the event(s)?”, “What were some barriers or challenges to delivering the COVID-19 vaccine in a non-clinical setting?”, and “What do you think could have made the event(s) more successful?” Based on the responses to the grand tour questions, additional probe questions were used based on CFIR categories and specific determinants. In addition, each participant was asked to discuss the extent to which the event(s) achieved the goal of reaching the communities of interest. All study materials and procedures were approved by the UAMS Institutional Review Board (IRB#262917).

### Data analysis

The co-authors conducted rapid thematic analysis following a modified framework approach (55, 56), utilizing CFIR as the *a priori* coding framework. Themes from each interview transcript were independently summarized by co-authors (GC, SP, JCK, and RM) using a structured coding template. The research team met regularly to consolidate the templates into one final coded template per interview, resolve discrepancies in interpretation, and assign identified barriers and facilitators to CFIR constructs. Barriers and facilitators were added to the operational definitions of constructs in the study-specific CFIR codebook. Summaries of coded data were transferred to charts with a column for each CFIR construct and a row for each participant to facilitate identification of patterns and outliers. The research team reached thematic saturation, e.g., the point at which patterns in the data were clearly identified through analysis and no new themes were identified, after analysis of 10 transcripts. Illustrative quotes were identified for each theme using a consensus approach.

## Results

Seventeen participants completed qualitative interviews (Table 1). Participants were healthcare providers (*n* = 4), program staff (many of whom were CHWs) (*n* = 10), and FBO staff (*n* = 3). The median age of participants was 41 years, and 65% of participants were women. Participants were racially/ethnically diverse; eight participants identified their race/ethnicity as White (47%), four identified as Hispanic (24%), four identified as Marshallese (24%), and one identified as Asian (6%).

**Table 1:** Demographics of participants (*n* = 17).

	Range	Median
Age, in years	23–55	41
	Frequency	Percent (%)^a^
Primary role		
Healthcare providers^b^	4	24%
Program staff^c^	10	59%
FBO staff	3	18%
Gender		
Woman	11	65%
Man	6	35%
Self-reported race/ethnicity		
White	8	47%
Hispanic	4	24%
Marshallese	4	24%
Asian	1	6%

^a^ May not equal 100% due to rounding.

^b^ Includes physicians, pharmacists, and nurses.

^c^ Includes various roles including event coordination, outreach, attendee registration, etc.

**Figure 1:**
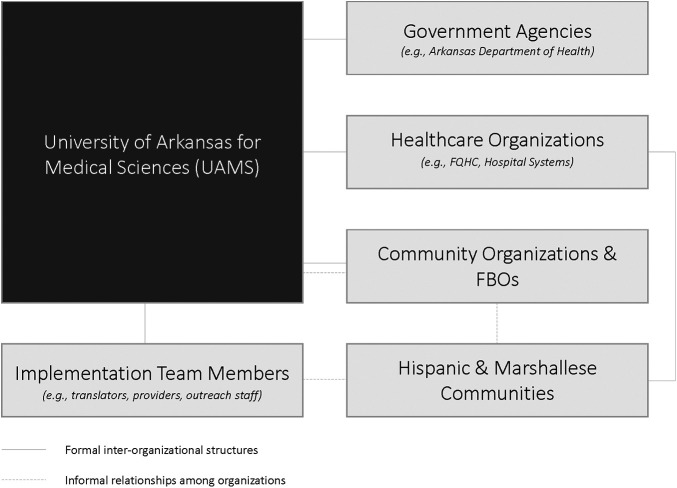
External relationships in the implementation context.

During analysis, we identified emergent themes within the *a priori* CFIR 1.0 category of “cosmopolitanism.” Participants described both formal and informal organizational-level relationships among UAMS and team members, government agencies {e.g., Arkansas Department of Health, the Consulate of the Republic of the Marshall Islands, healthcare organizations [e.g., Federally Qualified Health Centers (FQHCs), hospital systems]}, and community organizations and FBOs. We identified themes at the organizational and individual levels for external relationships as implementation determinants (see Table 2). At the organizational level, formal inter-organizational structures within the implementation environment and informal relationships among organizations emerged as implementation determinants. At the individual level, we identified themes of cultural congruence arising from the overlapping staff roles of the implementing organization with communities of focus and community members' familiarity with organization as determinants of community-based COVID-19 vaccination events. Formal inter-organizational structures included contractual agreements between organizations. Informal relationships among the implementing organization and partner organizations were developed over time through prior community-engaged collaboration (41, 47, 48). In addition, participants identified individual-level relationships, highlighting cultural congruence among community team members with dual roles in the implementing organization and as trusted members of the Hispanic or Marshallese communities. Cultural congruence, e.g., inclusion of community members as implementation team members, also facilitated community members' familiarity with the organization. The nature of external relationships, and their role as barriers and facilitators of community-based COVID-19 vaccination events, are described below highlighting the salient formal and informal relationships. Figure 1 presents an overview of the salient relationships within the implementation context.

**Table 2:** Emergent themes by level of relationship.

	Type of connection	Barrier	Facilitator
Organizational Level	Formal inter-organizational structures	• Mismatch of policies among organizations • Changing sponsors and rules at different events • Need for extensive coordination among and within organizations	• Regular communications • Formal institutional agreements (e.g., MOU, data use agreements) • Trust in partnerships among organizations
	Informal relationships among organizations	• Lack of pre-existing relationships with all relevant community organizations (e.g., some churches/FBOs were difficult to engage)	• Established communication channels and working relationships • Familiarity/trust between individuals
Individual Level	Staff roles in organization and community	• Burnout among bilingual staff and staff from communities of focus	• Provide culturally appropriate services in language • Intrinsic motivation of staff • Community members trust information provided by representative staff
	Community members’ familiarity with organization	• Some community members and sub-populations remain difficult to reach	• Community familiarity with UAMS in partnership with community organizations • Community members trust services offered by/at familiar organizations

### Organizational level: formal inter-organizational structures

The central inter-organizational structure for the intervention was a COVID-19 taskforce, described above. The community-led taskforce was organized as an extension of the community-engaged relationships among UAMS and team members, government agencies (e.g., Arkansas Department of Health), healthcare organizations (e.g., FQHC, hospital systems), community organizations and FBOs, and the Hispanic and Marshallese communities. The taskforce met monthly with an agenda focused on addressing COVID-19 disparities in the Hispanic and Marshallese communities. The taskforce was predicated on long-standing community-based relationships. Most taskforce members had established memoranda of understanding (MOU) or other formal agreements, such as data use agreements.

These formal relationships were identified by participants as facilitating implementation of the intervention in three key ways. First, the established weekly meetings of the formal partner organizations facilitated communication, serving as a forum for coordinating activities and providing input on intervention design. Second, pre-existing formal relationships (e.g., pre-existing MOU and data use agreements) among partner organizations enabled the quick start-up times for developing and deploying the vaccine events. One participant explained, “We keep getting in these projects, but it's not like we’re having to shift gears or do any 180s to do that work… these projects just allow us to continue, I guess, the work that we’ve already been doing, and enhance on it” (program staff #1, White). Third, the long-standing formal collaborations contributed to the trust among the partners in external networks. Explained by one participant, “We’ve worked with [community-based partner organizations] a lot in the past. They’ve always been a good partner, so it was kind of natural for us to go with them” (program staff #2, White).

Formal relationships were not uniformly discussed as facilitators, with some participants identifying barriers, such as differences in organizational policies which created operational challenges during events. For example, the categories of healthcare providers qualified to provide vaccinations varied by organization. One participant stated, “The most challenging thing about administering vaccines off-site is the regulation around it, [for UAMS supported events, the policy is] a credentialed provider [an MD, PA, or APRN] has to be on-site. Making sure that we're in compliance with all of the policies […] was probably the most challenging thing” (healthcare provider #2, Asian).

Additionally, the collaborative nature of the events meant variability in sponsors at some events which required changes in paperwork and electronic health records systems from one event to another, along with corresponding staffing and workflow changes. A team member stated, “When we first started partnering with [the FQHC], […] there's two consent forms they have to do, vs. the one when it's UAMS. […] At first, we didn’t have a good flow for when people came in and did the consent forms, and then got their shot and waited” (program staff #2, White). External relationships were also identified as requiring increased time and effort in communication and coordination by one participant: “I think definitely more communication between the partners [is important] so that everybody understands everybody else's roles and so when we start the event and we're there—again, to be flexible and culturally sensitive to who's there, understanding that not everything is gonna work like clockwork” (healthcare provider #1, Marshallese).

### Organizational level: informal relationships among organizations

Informal relationships across organizational boundaries were described as a primary facilitator at the organizational level and consisted of personal and professional connections among team members at the implementing organization and partner organizations (e.g., FBOs, government agencies, healthcare organizations). These informal relationships, described as developing over time, fostered collaboration across organizational boundaries during implementation of vaccination events. A leader at an FBO described how a connection between that organization's Executive Director and the Director of Health Outreach at a local hospital facilitated a vaccine clinic event. The participant stated, “We were talking to [Hospital] about that, and what all services they offered already […] and would they be willing to be our community provider” (FBO staff #2, White). That participant concluded, “So, we just facilitated it, and made it happen.”.

Other participants explained how long-standing informal relationships between organizations engendered trust and willingness to engage in implementing vaccination events. One participant noted, “It really helps already having the relationships there because we know that, ‘Oh, hey, I can work with this person from [a partner organization],’ and they’ve been a good partner in the past; we know they’re gonna show up if they say they’re gonna show up” (program staff #2, White). Conversely, there was greater difficulty in engaging organizations in vaccine events when informal relationships did not already exist. Participants noted that when trying to recruit new FBOs to participate in vaccination events, “there have been several churches that said, ‘We don't need ‘em’” (program staff #3, Marshallese).

### Individual level: staff roles in organization and community

As a result of long-term engagement with the Hispanic and Marshallese communities in Northwest Arkansas, several team members employed by UAMS are members of the Hispanic and Marshallese communities facilitating cultural congruence between the implementing organization, FBO, and the Hispanic and Marshallese communities. Participants overwhelmingly stated that cultural congruence among the implementing organization and communities of focus made it possible for team members at vaccine events to provide culturally and linguistically appropriate services. For example, one participant described culturally-appropriate messaging delivered by a trustworthy community ambassador as a facilitator for reaching the Marshallese community: “We're speaking with the Marshallese community, in Marshallese, in a way that's culturally appropriate by somebody that they trust” (healthcare provider #1, Marshallese). Team members' work in their communities was also considered a source of intrinsic motivation. One participant stated, “[Implementation team members are] part of the community that we’re protecting; the same people who come and work our events. […] So, they really take ownership […] sometimes they’re like, ‘Hey, this is my church. I’m gonna be there. I’m gonna work it. I’m gonna make sure it's successful’” (program staff #4, White).

Several Hispanic and Marshallese participants noted burnout resulting from their dual roles as UAMS team members and as members of the communities of focus. Participants described how this overlap in social roles became an implementation barrier as events required the involvement of bilingual team members at every stage of the process (check in, registration, consent, vaccination, waiting period, and evaluation activities). Team members with the requisite language skills worked additional and non-standard hours to accommodate vaccination events which occurred on nights and weekends and in varying locations in the region. Furthermore, some Hispanic and Marshallese staff reported serving as an unofficial point-of-contact or source of information for members of their own community outside of their work hours. A participant described, “'Cause I work with UAMS, they assume that I have the answer when they ask me things [about COVID-19 or the vaccine]” (program staff #6, Marshallese). Institutional policies related to flexible hours and paid time off were described by participants as critical to helping mitigate this barrier. One participant explained, “You get—we call it flex time. Say you worked five hours on an event on Sunday. You can then take those five hours off somewhere else in the next two weeks during your regular work time, without having to submit time through the system. That works well, except everybody's very busy, and needs their office time too” (program staff #5, Hispanic).

### Individual level: community members' familiarity with organization

Participants frequently described how Hispanic and Marshallese community members in Northwest Arkansas were familiar with UAMS due to the organization's prior community-based and community-engaged research, programs, and outreach. Participants described community members' familiarity with UAMS, and the employment of staff from their communities, as facilitating reach: “Many of the Marshallese events are being organized by Marshallese folks [staff]. They know of us, so when we approach them—I don't think anybody has turned us down yet” (healthcare provider #1, Marshallese). Long-term community engagement and outreach was described as building trust, supporting the sustainability of external relationships, and improving community buy-in with the vaccine events. As one participant narrated, “I moved here about five years ago, and I saw some of my colleagues work out in the community. […] I was impressed with the work they were doing out in the community. So, I said to myself, well, I'm gonna apply there ‘cause I wanna do work there. I wanna be among those people that are doing impact work with the community” (program staff #6, Marshallese).

In spite of the facilitating role of familiarity with UAMS, participants identified limits to reach stemming from unmet communication needs, especially for some sub-populations who were not as easily reached, even by bilingual Hispanic and Marshallese team members. One participant specifically mentioned reaching older members of the Hispanic community as a barrier: “I have noticed that most of the elderlies [from the Hispanic community] that we get vaccinated, it's somebody else who's bringin’ them. It is not them who got the information firsthand.” This participant also stated that low literacy among older members of the Hispanic community was not addressed through normal outreach techniques: “We still give them the [printed flyers] but there is also the fact that usually Hispanic populations […] our elderlies, […] most of them don't know how to read or write” (program staff #7, Hispanic).

## Discussion

This paper explored external relationships (organizational and individual) as barriers and facilitators for equitable community-based implementation of a COVID-19 vaccination intervention leveraging FBOs to reach underserved and hard-to-reach Hispanic and Marshallese communities. Engagement with FBOs to promote health equity and mitigate health disparities among Hispanic and/or Marshallese communities is described in detail in prior publications (41, 49, 57–60). Importantly, vaccination events held in partnership with FBOs reached a higher proportion of Hispanic and Marshallese persons compared to vaccination events in secular, community contexts, and individuals vaccinated at these events were more likely to report completely trusting the COVID-19 vaccine (49). We identified themes at the organizational and individual levels for external relationships as implementation determinants. At the organizational level, formal inter-organizational structures and informal relationships among organizations, which predated the development and implementation of the community-based COVID-19 vaccine events, emerged as implementation determinants. At the individual level, implementation determinants included staff roles in organization and community and community members' familiarity with organization. Participants also identified team members who belonged to the communities of focus as a facilitator for vaccination events, especially for providing culturally appropriate services in language and for leveraging cultural congruence and community members' familiarity with the implementing organization to improve reach. Participants described external relationships as critical factors in creating and maintaining trust among partner organizations and within the Hispanic and Marshallese communities.

Across organizational and individual levels, our analysis highlights three critical factors among organizational and interpersonal relationships as implementation determinants. First, cultural congruence of the implementation team with target communities can be critical to implementing equitable community-engaged interventions. Our findings highlight how implementing organizations can leverage cultural congruence among implementation team members and communities of focus to facilitate outreach, build and maintain trust, and improve the reach of interventions into underserved and hard-to-reach communities. These team members served as an important intermediary between healthcare organizations, community partner organizations, and community members (14, 17, 61–64). Community representation among healthcare workers has also been reported to build trust among communities and healthcare actors (65, 66). However, these team members face unique pressures from overlapping social roles which intertwine their personal and professional lives and may result in greater levels of stress, emotional exhaustion, and burnout (67, 68), particularly during health emergencies that disproportionately impact their communities, such as the COVID-19 pandemic. Future studies of the role of organizational relationships in implementation should consider the interaction between these relationships and “characteristics of individuals” involved in implementation (4), particularly the needs, capability, and motivation of individuals with dual roles in the implementing organization and the community of focus.

Second, long-term, pre-existing relationships among organizations and individuals facilitated implementation and outcomes. Specifically, the preexisting investment in community engagement facilitated the rapid development and implementation of the vaccine events. While it is well-established that building strong relationships is often an essential step in successful community-based interventions, building these relationships with communities requires time and focused effort, even when sufficient organizational resources are available (69, 70). Time, as a contextual variable in implementation, is not explicitly addressed by most IS frameworks, and it is often reduced to a static resource (3). Our findings suggest that the positive effects of community engagement and trust-building strengthen over time, with long-standing formal and informal relationships described most frequently as a critically-important facilitator. The role of time in strengthening community and organizational relationships contrasts with the dynamics of time in the Stages of Implementation Completion framework, where longer durations for tasks predict poor implementation. This gap highlights the lack of focus on community engagement and organizational relationship-building as ongoing processes and implementation determinants (71, 72). However, CEnR scholars, including Wallerstein and colleagues, have cited the importance of long timeframes noting that “despite enhanced focus on research and health outcomes” in community-engaged participatory projects, “the science lags behind the practice,” with little evidence on the mechanisms through which engagement results in outcomes (37). The CEnR literature, especially for community-based participatory approaches, focuses more explicitly on time as a critical factor, and this body of work could be drawn upon to inform future research on community-engaged implementation (35, 73).

Finally, our findings also have implications for trust and trustworthiness as important constructs in equity-focused, community-engaged implementation. Participants explained how long-standing informal relationships among target communities and organizations involved in the intervention engendered trust in new activities or programs facilitating the involvement of hard-to-reach populations, which is broadly consistent with the community engagement literature (37, 69, 74–76). Long-term community engagement also contributed to organizational capacity for culturally-appropriate interventions, as well as the recruitment and retention of community staff whose contributions to COVID-19 vaccine events were described as essential factors in the success of the events through cultural congruence which created and reinforced trust among Hispanic and Marshallese individuals who were more comfortable with members of their own community (74, 77–79).

Recent calls for IS to focus on health equity and adopt justice-focused approaches requires an increased focus on organizational relationships and community engagement, and implementation researchers could draw on the robust CEnR literature to improve health equity (73, 77, 80, 81). Addressing structural health inequalities at the intersections of race, gender, sexual orientation, and immigration status will require a greater focus on incorporating interested communities and individuals into the development, implementation, and evaluation of interventions (27, 28, 32, 82, 27). Partnership strategies have been found to help mitigate and reduce inequities in care, promote individual empowerment, and reduce social stigma of health conditions, which further supports the results of the present study (77, 80). Our findings suggest that if implementation researchers are to engage with communities to mitigate health disparities and promote health equity, attention needs to be paid to both formal and informal relationships among academic institutions, healthcare systems, healthcare providers and clinics, community-based organizations, and communities.

### Strengths and limitations

This exploratory study was limited to Northwest Arkansas and focused on COVID-19 vaccination promotion to the Hispanic and Marshallese communities, and hence, generalizability may be limited. Further, our methods were solely qualitative in nature, which also limits generalizability of the findings. These limitations are offset by the diversity and rigor of the qualitative methods used and by the experience and expertise of the research team. As well, the relationships among organizations and the Hispanic and Marshallese communities under study were large and well-established, which provided an opportunity to investigate the potential longer-term implementation-related impacts of these relationships.

## Conclusion

Achieving health equity in IS requires a greater understanding of external relationships, both at the organizational and individual levels, as implementation determinants; however, there are significant gaps in the current understanding of how these relationships affect implementation. This article makes a significant contribution to the literature through our exploration of formal and informal relationships among organizations and individuals as critical implementation determinants for community-based COVID-19 vaccination events within FBOs as a way to reach underserved and hard-to-reach Hispanic and Marshallese community members. Across all levels of our analysis, we identified three critical factors for external relationships as implementation determinants. First, cultural congruence of the implementation team with communities of focus can be leveraged to facilitate outreach, build and maintain trust, and improve the reach of interventions into underserved and hard-to-reach communities. Second, long-term, pre-existing relationships allowed for rapid implementation. This study expands the current literature on time as a contextual implementation determinant, which is underdeveloped in IS, with our findings suggesting that the positive effects of community engagement and trust-building strengthen over time and facilitate subsequent outreach and interventions. Finally, this study has implications for understanding trust and trustworthiness in equity-focused, community-engaged implementation, with long-term community engagement contributing to organizational capacity for culturally- and linguistically-appropriate interventions. While these concepts are understudied in IS, the literature of CEnR may be useful in informing IS. Further research is needed to clarify and understand the precise effects of external, organizational relationships on other implementation determinants.

## Data Availability

The deidentified data underlying the results presented in this study may be made available upon reasonable request from the corresponding author, PM, at pamcelfish@uams.edu.
