# Supplementary material for: External relationships as implementation determinants in community-engaged, equity-focused COVID-19 vaccination events
Source: Front Health Serv. 2024 Mar 11;4:1338622. doi: 10.3389/frhs.2024.1338622 (PMC10964718; doi:10.3389/frhs.2024.1338622)
Supplement: Supplementary file 1 [file Datasheet1.docx]

| **COVID-19 Vaccine Implementation Interview Guide:**  **Staff and Volunteer** |
| --- |

**An Ethnographic Study of COVID-19 Vaccine Implementation Audio Recorded Qualitative Questions**

My name is __________________ thank you for participating in this interview. I (we) want to ask you a few questions regarding the Covid-19 vaccine event(s) you participated in.

**Main Questions:**

What was your role at the event(s)?

Paid? Volunteer? Affiliated with a community organization? UAMS?

How did you come to work at the COVID-19 vaccine community event(s)?

Probe reasons for staffing at the event.

How would you describe your experience at the COVID-19 vaccine event?

What do you think worked well at the event?

What do you think could have been done better at the event?

What were some barriers or challenges to delivering the COVID-19 vaccine in a non-clinical setting (i.e., at a church or community-based location)?

What were some elements (materials, people, timing, etc.) that made delivering the COVID-19 vaccine in a non-clinical setting possible?

What do you think could have made the event more successful?

***Probe: Do you feel that the event reached the target population?***

***Probe: How could we make delivering vaccines or other medical interventions easier for the community to host and/or attend?***

What do you think could be done to make vaccine or other medical intervention delivery common place at your organization?

Do you have any recommendations for adapting vaccine delivery or other medical intervention events to better fit the community’s needs?

***Probe: Are there any specific actions that UAMS or community organizations could take to make the vaccine events more successful?***

**Closing Question**

Is there anything about the COVID-19 vaccination event that you would like to share or talk about that was not asked?
